# Disparities in Criminal Sanctions and the Role of Non-Formal Environmental Education in the Enforcement of Corporate Waste Law: A Comparative Study of Two Tangerang District Court Rulings

**DOI:** 10.12688/f1000research.187336.1

**Published:** 2026-08-05

**Authors:** Ira Sinta Azlina, Andre Arya Pratama, Moh. Hariyanto, Reski Hasan, Putra Ramadhan, Nisa Gustiara, Nur Fauzi Ramadhan

**Affiliations:** 1 Faculty of Law, Gadjah Mada University Faculty of Law, Yogyakarta, Special Region of Yogyakarta, 55281, Indonesia; 2 Non-Formal Education, Universitas Negeri Yogyakarta, Yogyakarta, Special Region of Yogyakarta, 55281, Indonesia; 3 Faculty of Law, University of Indonesia Faculty of Law, Depok, West Java, 16424, Indonesia

**Keywords:** Keywords: environmental criminal law; corporate criminal liability; hazardous and toxic waste (B3); illegal waste import; tragedy of the commons; Indonesia

## Abstract

**Background:**

International attention to Indonesia’s exposure to the global waste trade has grown since China’s National Sword policy (2018) diverted waste flows to Southeast Asia. Indonesian legal scholarship has largely focused on administrative/civil liability or Tragedy of the Commons theory, while empirical case studies grounded in verifiable primary rulings remain scarce, as does attention to education as a preventive complement to criminal sanctions. This study addresses both gaps through a comparative case study of two corporate environmental criminal cases decided by the Tangerang District Court.

**Methods:**

A qualitative legal case study using documentary technique on two rulings: Tangerang District Court Ruling No. 1834/Pid.Sus/2020/PN Tng (PT Indonesia Toray Synthetics, verbatim primary source) and Ruling No. 1606/PID.B/LH/2022/PN TNG (PT Advance Recycle Technology, reconstructed from the prosecutor’s statement and cross-verified through four media outlets). Data were analyzed against Law No. 32/2009 and the theories of Tragedy of the Commons and Unequal Ecological Exchange.

**Results:**

PT Indonesia Toray Synthetics was proven to have dumped hazardous waste (B3) without a permit (Articles 60/104) and was fined Rp300 million plus a clean-up obligation. PT Advance Recycle Technology was proven to have imported waste across the border without a permit (Articles 105/106/116/118–119) and was fined Rp4 billion with no recorded additional sanction. An inverse relationship emerged between fine size and the presence of a remedial sanction.

**Conclusion:**

Indonesian courts can impose meaningful criminal sanctions, but the Article 119 remedial sanction is applied inconsistently, particularly in the larger cross-border case. Recommended reforms include standardizing remedial sanctions, strengthening cross-border verification, improving public access to rulings, and expanding non-formal environmental legal education (Article 65(2)) to address the detection gap revealed by both cases.

## Introduction

China’s National Sword policy, enforced since early 2018, closed the world’s largest waste import market to 24 categories of solid waste a market that had previously absorbed 72.4 percent of the volume of used plastic traded globally ( Brooks, Wang & Jambeck, 2018). The diversion of waste flows to Southeast Asia resulting from this policy increased the volume of Indonesia’s waste imports by around 141 percent in 2018 alone, with imported plastic buildup clearly visible in Mojokerto, Sidoarjo, and along the Brantas River watershed ( Ismawati et al., 2024).

This phenomenon has been widely analyzed in Indonesian legal scholarship through the framework of Garrett Hardin’s 1968 essay, The Tragedy of the Commons, as well as the Unequal Ecological Exchange (UEE) theory developed by Martínez-Alier (2003) and Hornborg (1998), which explains the transfer of ‘ecological debt’ from rich countries to poor countries as a structural feature of the global economy, rather than merely an incidental market failure. What has been relatively less examined is how this theoretical proposition manifests in Indonesian criminal cases that have actually been decided by courts not as illustrative anecdotes drawn from media coverage, but as findings verifiable from primary ruling sources.

This study addresses that gap by examining two corporate environmental criminal cases decided by the same court, the Tangerang District Court, which sits within the industrial corridor most affected by the post-2018 surge in waste imports. The first case concerns PT Indonesia Toray Synthetics, which was found guilty of disposing of hazardous and toxic waste (B3) outside its licensed storage area at its own production facility. The second case concerns PT Advance Recycle Technology, which was found guilty of importing waste across Indonesia’s border without the required permit. Read together, these two cases cover two faces of the waste-trade issue outlined above one domestic and the other cross-border allowing this study to ask, with case-level precision, whether Indonesia’s criminal courts impose sanctions consistently on both faces of this same underlying issue.

The relevance of this comparison has increased further since the Minister of Environment of the Republic of Indonesia imposed a total ban on plastic scrap imports in January 2025, replacing the previous 2 percent contamination threshold that had proven difficult to enforce ( Mongabay Indonesia, 2025). The effectiveness of this administrative ban will depend heavily on whether the criminal justice system is able to apply consistent, proportionate, and remedial sanctions. Beyond the criminal-law findings themselves, this study also asks what a community-oriented preventive complement might look like, given that both cases were detected solely through institutional inspection, not through community reporting.

## Problem statement

The existing doctrinal literature on Indonesia’s exposure to international waste trade tends to rely on aggregate statistics (tonnage, container counts) and normative criticism of the Basel Convention’s enforcement architecture, without in-depth, source-verified examination of how individual criminal cases are actually tried and sanctioned. This creates a gap between the urgency depicted for this phenomenon and the depth of scholarship on its primary legal remedy criminal adjudication using verifiable primary sources.

This gap has practical consequences. Without case-level evidence on how the mechanism for shifting liability to the corporation under Article 116 and the remedial sanction under Article 119 are actually applied in practice, policymakers and prosecutors have little empirical basis to assess whether current sentencing practice is proportionate to the scale of harm caused, or whether it is applied consistently to comparable violations.

## Research objective

This study aims to: 1) reconstruct, using verifiable documentary sources, the chronology and sanctioning outcomes of two corporate environmental criminal cases decided by the Tangerang District Court; 2) compare the elements of liability proven and the sanctions imposed in each case against the framework of Law No. 32 of 2009; and 3) interpret this comparison through Hardin’s Tragedy of the Commons and Unequal Ecological Exchange theories, in order to formulate recommendations for more consistent criminal law enforcement against corporate waste offenses in Indonesia.

## Literature review

### Pollution as a reverse tragedy of the commons

Hardin (1968) distinguished the depletion of a shared resource (taking from the commons) from its pollution (adding to the commons), arguing that under the second mechanism, a rational actor’s individual cost-benefit calculation will favor externalizing waste onto a shared space whose capacity is assumed to be free and ownerless. When the receiving jurisdiction has weaker monitoring capacity, this calculation becomes increasingly advantageous for the polluter, regardless of that jurisdiction’s formal territorial sovereignty.

### Unequal ecological exchange

Drawing on Wallerstein’s (2004) world-systems analysis, Martínez-Alier (2003) and Hornborg (1998) argue that the global capitalist economy systematically shifts biophysical burdens including waste, emissions, and ‘ecological debt’ from core countries to periphery countries. Givens, Huang, and Jorgenson (2019) note that a periphery country’s limited legal, technical, and diplomatic capacity is not incidental to this dynamic, but structurally makes it a rational destination for waste that wealthier countries do not wish to process themselves.

### Corporate criminal liability in Indonesian environmental law

Law No. 32 of 2009 on Environmental Protection and Management (UUPPLH) is the principal criminal-law basis for environmental law enforcement in Indonesia. Table 1 sets out the verbatim text of seven articles relevant to the two cases examined in this study, quoted directly from the official consolidated text of the Law published by the legal documentation center of the Ministry of Energy and Mineral Resources (JDIH ESDM, 2009).

**Table 1.** Article framework: verbatim text of Law No. 32 of 2009 relevant to both cases, quoted from the official consolidated text (JDIH ESDM, 2009).

Article	Verbatim text (excerpt)	Relevance to this study
Article 60	“Every person is prohibited from dumping waste and/or materials into the environment without a permit.”	Basis for Case 1 (Toray): dumping of hazardous and toxic waste (B3) without a permit outside the designated storage area
Article 104	“Any person who dumps waste and/or materials into the environment without a permit as referred to in Article 60 shall be sentenced to a maximum of 3 (three) years’ imprisonment and a maximum fine of Rp3,000,000,000.00.”	The criminal sanction actually imposed in Case 1 (a fine of Rp300 million, still within the statutory maximum)
Article 105	“Any person who brings waste into the territory of the Unitary State of the Republic of Indonesia as referred to in Article 69 paragraph (1) letter c shall be sentenced to a minimum of 4 (four) years’ and a maximum of 12 (twelve) years’ imprisonment and a minimum fine of Rp4,000,000,000.00 and a maximum of Rp12,000,000,000.00.”	One of two alternative charges against PT Advance Recycle Technology in Case 2
Article 106	“Any person who brings hazardous and toxic waste (B3) into the territory of the Unitary State of the Republic of Indonesia as referred to in Article 69 paragraph (1) letter d shall be sentenced to a minimum of 5 (five) years’ and a maximum of 15 (fifteen) years’ imprisonment and a minimum fine of Rp5,000,000,000.00 and a maximum of Rp15,000,000,000.00.”	Alternative charge against PT Advance Recycle Technology in Case 2; the statutory maximum fine (Rp15 billion) serves as the benchmark for assessing the Rp4 billion fine actually imposed
Article 116	“If an environmental crime is committed by, for, or on behalf of a business entity, criminal prosecution and criminal sanctions shall be imposed on: a. the business entity; and/or b. the person who gave the order… or the person acting as the leader of the activity.”	Basis for shifting liability to the corporation, used in Case 1 and Case 2
Article 118	“For the criminal offense as referred to in Article 116 paragraph (1) letter a, the criminal sanction shall be imposed on the business entity, represented by the management authorized to represent it in and out of court… as the functional perpetrator.”	The mechanism whereby a company director (Case 1) formally represents the corporate defendant
Article 119	“In addition to the punishment referred to in this Law, a business entity may be subjected to additional punishment or disciplinary measures in the form of: a. confiscation of profits…; b. closure of the whole or part of the place of business…; c. remediation of the consequences of the criminal act; d. an obligation to carry out what was neglected without right; and/or e. placement of the company under guardianship for a maximum of 3 years.”	Legal basis for the additional clean-up sanction in Case 1 (letter c); no equivalent additional sanction reported in Case 2

**Table 2.** Comparative summary of the two cases.

Aspect	Case 1: PT Indonesia Toray Synthetics	Case 2: PT Advance Recycle Technology	Analytical implications
Source status	Primary - verbatim ruling text (Supreme Court Ruling Directory)	Secondary - prosecutor’s statement confirmed by 4 news sources	Case 1’s findings carry greater evidentiary weight; Case 2’s findings are presented with an explicit source marker
Type of offense	Domestic unlicensed dumping of hazardous and toxic waste (Articles 60/104)	Unlicensed cross-border waste import (Articles 105/106/116)	Both cases capture two distinct nodes of the same ‘reverse commons’ dynamic: domestic mismanagement and international transfer
Fine imposed	Rp300 million	Rp4 billion	A 13x difference, roughly proportionate to the scale of the violation, but not matched by an equivalent difference in remedial sanctions
Additional sanction (remedial)	Yes - obligation to clean up and hand over to a licensed manager, at the company’s expense	Not reported	The larger cross-border violation did not, in fact, receive a recorded remedial order
Ruling date	9 August 2021	15 December 2022	Both occurred after the 2018 National Sword surge, placing both cases within the same wave

## Method

This study uses a qualitative legal case study design following Yin’s (2018) conception of case study research as an empirical inquiry into a contemporary phenomenon within its real-life context, with documentary technique as the data collection method suited to legal case studies (rather than fieldwork-based methods). Two units of analysis were selected: Tangerang District Court Ruling No. 1834/Pid.Sus/2020/PN Tng, obtained in full from the Supreme Court Ruling Directory and quoted verbatim throughout this article; and Tangerang District Court Ruling No. 1606/PID.B/LH/2022/PN TNG, whose complete primary text could not be obtained through the same directory at the time of writing, and which was therefore reconstructed from the official statement of the Tangerang City District Attorney’s Office on the case outcome, cross-verified through four independent news sources to ensure internal consistency of the case number, the articles charged, and the size of the sanction.

The data were analyzed using the condensation-display-verification logic adapted from the interactive qualitative analysis model of Miles, Huberman, and Saldaña (2014), applied here to documentary data rather than interview or observational data; relevant facts were nonetheless extracted from each source, cross-checked for consistency, arranged into a case chronology and a thematic comparison table, and interpreted against the normative elements of Article 60 and Articles 104–106 as well as Articles 116–119 of Law 32/2009, and against the theoretical propositions reviewed above. Every empirical claim in the Results section below is accompanied by explicit source attribution within the text (bodynote), and any claim resting on the secondary-sourced case (PT Advance Recycle Technology) is explicitly flagged as such.

## Result and discussion

### Case 1 - PT indonesia toray synthetics

Based on the verbatim text of Tangerang District Court Ruling No. 1834/Pid.Sus/2020/PN Tng, PT Indonesia Toray Synthetics, a producer of synthetic fiber and resin located at Jalan Mochamad Toha KM.1, Karawaci, Tangerang City, was charged after hazardous and toxic waste (B3) was found stored outside the designated Temporary Storage Area (TPS) specifically, in an area adjacent to the company’s Fly Ash TPS and Non-B3 TPS ( Supreme Court Ruling Directory, n.d.). The case file shows that the company essentially held a complete and valid set of environmental permits, including an Environmental Permit (2015), a Wastewater Discharge Permit (2015), and two Hazardous Waste Management Permits for temporary storage (2016), as well as a national-level Hazardous Waste Utilization Permit issued by the Ministry of Environment and Forestry in 2017 meaning that the charged offense was a location- and procedure-based violation within a waste management system that was, in essence, already licensed, rather than a total absence of permits ( Supreme Court Ruling Directory, n.d.).

On 9 August 2021, a panel of judges found the corporation, represented by its Director of the Production Division, legally and convincingly guilty of dumping waste and/or materials into the environment without a permit as referred to in Article 60 in conjunction with Article 104 of Law 32/2009 ( Supreme Court Ruling Directory, n.d.). The court imposed a fine of Rp300 million, with a provision that the company’s assets could be seized and auctioned if the fine were not paid within 30 days, and crucially an additional sanction consisting of an obligation to clean up the misplaced B3 waste, relocate it to a licensed storage area, and subsequently hand it over to a licensed third-party waste manager at the company’s own expense ( Supreme Court Ruling Directory, n.d.).

### Case 2 - PT advance recycle technology (secondary source, cross-verified)

The following reconstruction rests on the official statement of the Tangerang City District Attorney’s Office confirming payment of the fine in accordance with the court ruling (as reported by VOJ.co.id, 2023, and Koran Pelita, 2023), combined with Mongabay Indonesia’s 2019 investigative coverage of the case’s origins ( Mongabay Indonesia, 2019a, 2019b). The complete primary text of the ruling could not be obtained through the Supreme Court Ruling Directory at the time of writing; this limitation is discussed further in the following section.

PT Advance Recycle Technology was established in January 2018 and obtained Bonded Zone status in Tangerang in March 2019 ( VOJ.co.id, 2023). Following a request from the Tangerang Customs Office for a joint inspection of an imported plastic scrap shipment, the Ministry of Environment and Forestry (KLHK) declined to declare the shipment compliant ( Mongabay Indonesia, 2019a). In October 2019, the KLHK’s Directorate General of Law Enforcement named two Singaporean nationals the company’s commissioner and director as suspects under Article 105/106 in conjunction with Article 116 paragraph (1) letter a in conjunction with Articles 118–119 of Law 32/2009, calling it the first-ever naming of a foreign national suspect under the UUPPLH specifically for unlicensed waste import ( Mongabay Indonesia, 2019a). Nationally, the KLHK inspected 882 containers of plastic and paper scrap between April and August 2019, of which 87 containers of B3-contaminated plastic waste originating from Hong Kong, Spain, Canada, Australia, and Japan were found to have entered illegally ( Mongabay Indonesia, 2019b), demonstrating that this broader pattern formed the context for this case.

According to the prosecutor’s statement, the investigation established that the company held neither a Producer-Importer Identification Number (API-P) nor an Import Approval (PI), both of which are mandatory under Law No. 7 of 2014 on Trade and Minister of Trade Regulation No. 31 of 2016, and that it failed to meet the 90-day re-export obligation set out in Article 19 of that regulation for B3-contaminated shipments ( VOJ.co.id, 2023). On 15 December 2022, the Tangerang District Court found the corporation guilty under Article 105 in conjunction with Article 116 paragraph (1) letter a in conjunction with Article 118 in conjunction with Article 119, or alternatively Article 106 with the same accompanying articles, of Law 32/2009 (Tangerang District Court Ruling No. 1606/PID.B/LH/2022/PN TNG), and imposed a fine of Rp4 billion, which was paid by the company’s General Manager and confirmed by the Tangerang City District Attorney’s Office on 15 February 2023 ( VOJ.co.id, 2023; Koran Pelita, 2023). No additional (non-monetary) sanction was reported in these mutually corroborating sources, and the final outcome of the prosecution against the individual suspects could not be found in the public record available for this study.

### Comparative analysis

Figure 1 summarizes this comparison visually: Panel A places the two rulings on a timeline relative to China’s 2018 National Sword policy; Panel B compares the fine amounts in each case; and Panel C shows the presence or absence of a (non-monetary) remedial sanction alongside the monetary fine. Figure 2 then traces the law-enforcement pathway followed in each case, from initial administrative detection to the final criminal sanction, highlighting points of convergence and divergence between the two pathways.

**Figure 1.**
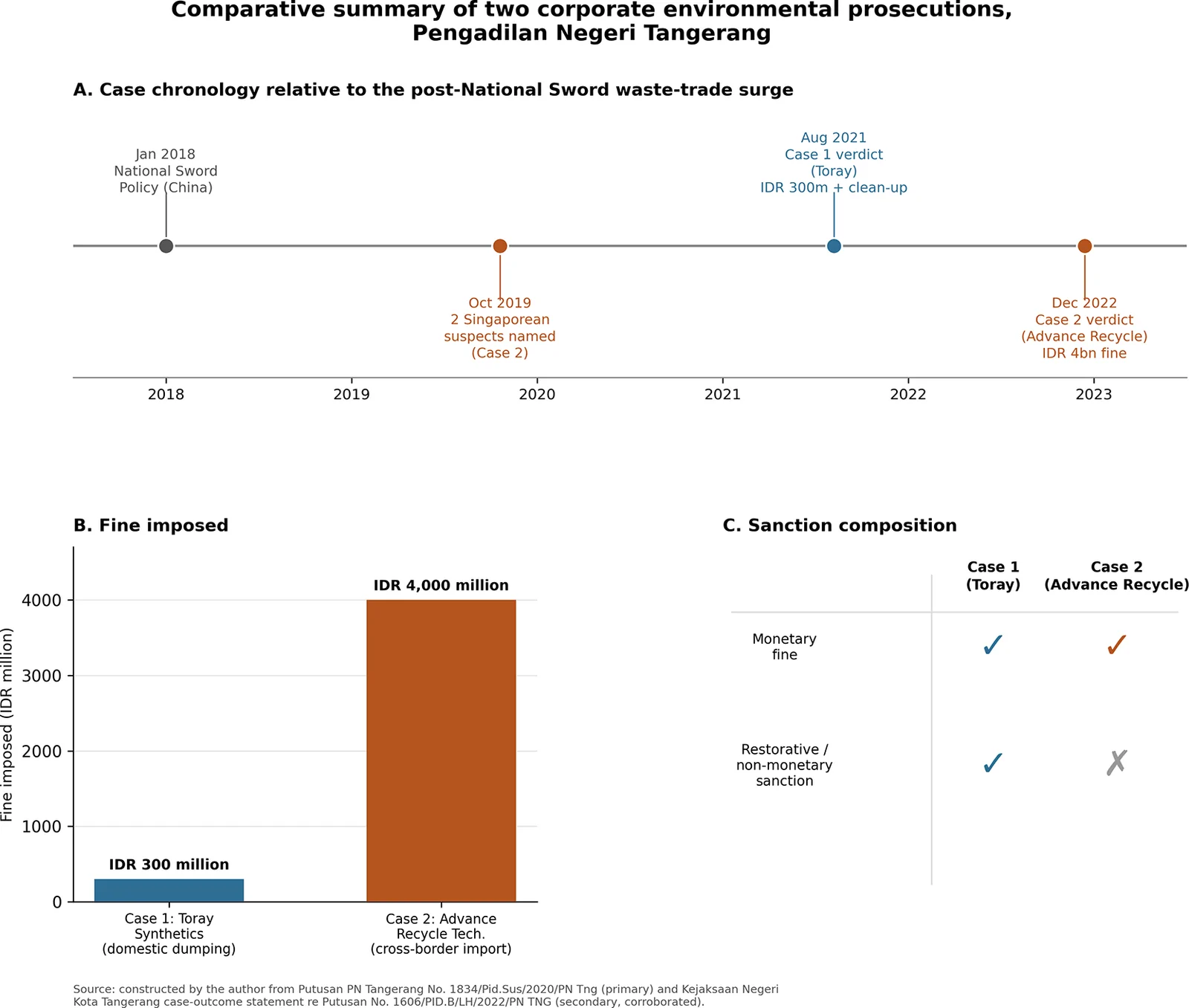
Comparative summary of the two corporate environmental criminal cases: (A) case chronology, (B) fines imposed, (C) sanction composition.

**Figure 2.**
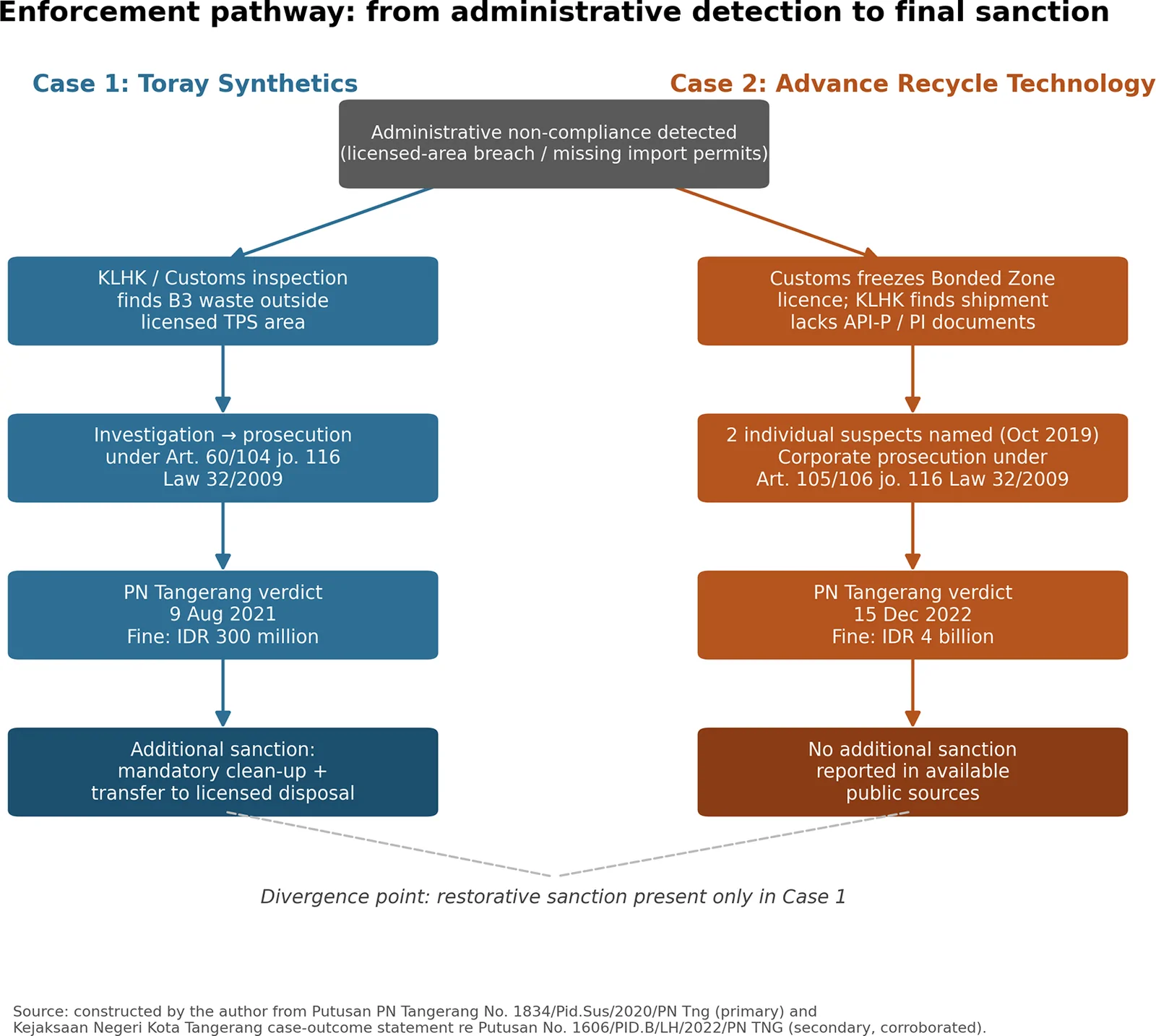
Flow diagram of law enforcement tracing each case from initial administrative detection to final sanction.

### Theoretical interpretation

Both cases are consistent with Hardin’s account of pollution as a reverse tragedy of the commons. In each case, the corporate actor’s cost calculation favored externalizing waste whether by storing it outside a licensed and monitored area (Toray) or by importing it without the documentation that should have required a compliant source (Advance Recycle Technology) onto a shared space whose capacity was treated as costless. The clean-up order in the Toray case represents a direct, court-ordered internalization of that externalized cost precisely the kind of ‘coercive device’ that Hardin argued was necessary because ‘the long-term costs of relying on conscience alone are reason enough to reject it’ as a primary regulatory strategy.

Only the Advance Recycle Technology case directly embodies Unequal Ecological Exchange in its cross-border form: a corporate management controlled by foreign parties, a Bonded Zone facility established just weeks after China’s National Sword policy, and waste originating from a range of wealthier trading partners (Hong Kong, Spain, Canada, Australia, Japan) all consistent with the biophysical transfer of ecological debt described by Martínez-Alier and Hornborg. The Toray case, by contrast, illustrates the domestic face of the same underlying logic: a foreign-affiliated producer generating B3 waste domestically and mismanaging its disposal, without any cross-border transfer element.

The most striking finding from this comparison is the inverse relationship between the size of the fine and the presence of a remedial sanction. If deterrence theory predicts that sanctions should be proportionate to the harm caused and the gain avoided, the larger cross-border violation should have received a remedial order at least as strong as the smaller domestic violation. The fact that this did not happen suggests that the application of the Article 119 remedial sanction in Indonesian environmental adjudication may depend more on case-specific factors (such as how easily a concrete remediation task can be identified, as with the on-site clean-up in the Toray case) than on a systematic policy that aligns the severity of the sanction with the scale of the violation.

## Implementation limitations and recommendations

Two limitations bear directly on the strength of this study’s claims. First, the evidentiary basis for Case 2 is secondary; although the case number, the articles charged, the size of the fine, and the payment date are confirmed identically between the prosecutor’s statement and independent news sources, this study lacks access to the panel of judges’ own written reasoning, so any claim about that case carries less evidentiary weight than an equivalent claim about Case 1. Second, as a comparison of two cases, this study’s findings depict a pattern worth further investigation rather than a statistically representative sample of environmental sentencing in Indonesia; a systematic multi-case study, ideally limited to cases whose primary texts can be obtained in full, is a natural extension of this study.

On this basis, four recommendations are proposed.

• Standardize the application of the Article 119 remedial sanction. Public prosecutors should demand, and courts should make a standard companion to any fine, a remediation order, licensing consequences, or corporate guardianship in every proven case of environmental dumping or illegal import not only when a concrete clean-up task happens to be easy to identify.
• Improve public accessibility to the full text of rulings. The Supreme Court Ruling Directory succeeded in providing the full text of Case 1 for this study; consistent indexing and searchability across all environmental criminal cases, including Case 2, would substantially strengthen the evidentiary base available for future case study research and for public accountability efforts.
• Reverse the burden of proof for waste classification at the border. Importers, rather than customs or environmental authorities, should bear the burden of proving that a shipment meets the applicable purity and classification standards before release, in order to reduce reliance on post hoc prosecution as documented in Case 2.
• Publish consolidated case-level law-enforcement statistics broken down by sanction type. Reporting not only the number of prosecutions and total fines, but also the incidence of remedial sanctions, would enable the kind of proportionality analysis attempted in this study to be conducted systematically, rather than case by case.

### Non-formal environmental legal education as a preventive complement

Both cases were identified through institutional inspection environmental compliance monitoring by the KLHK in Case 1, and a joint KLHK-Customs inspection request in Case 2. Law enforcement in both cases therefore depended entirely on the capacity and initiative of government oversight bodies, with no visible role played by communities living near the industrial facilities and bonded zones where the violations occurred. This suggests that one potential complementary layer of detection remains underused, and Indonesian law in fact already provides an explicit basis for developing it. Article 65 paragraph (2) of Law No. 32 of 2009 establishes that “every person has the right to environmental education” as part of the right to a good and healthy environment, and Article 63 assigns the central and regional governments the duty to provide “education, training, guidance, and awards” in environmental management. Indonesian environmental law scholarship interprets this provision as covering non-formal channels such as outreach, information dissemination, and non-formal legal education, alongside formal education in schools, as part of a preventive strategy that complements repressive law enforcement ( Solikin, 2019).

Table 3 summarizes this legal basis together with the types of non-formal environmental legal education already documented in practice in Indonesia. Expanding programs of this kind specifically to communities and workers around the Bonded Zone and industrial corridor (the same locations as both cases in this study) would not change the criminal-law recommendations above, but would address the detection gap that emerged independently from both cases.

**Table 3.** Legal basis and documented practice of non-formal environmental legal education in Indonesia, as a preventive complement to the criminal-law recommendations above.

Legal/academic basis	Content	Relevance
Law No. 32/2009, Article 65(2)	“Every person has the right to environmental education, access to information, access to participation, and access to justice in fulfilling the right to a good and healthy environment.”	Legal basis for treating environmental legal education as a citizen’s right, not merely an optional government program
Law No. 32/2009, Article 63	Assigns the central and regional governments the duty to provide “education, training, guidance, and awards” as part of their authority over environmental management	Places an affirmative obligation on government (at both the national level and the Tangerang city/provincial level) to run programs of this kind, rather than merely demanding compliance after a violation occurs
Solikin (2019), Kanun Journal of Legal Studies	Environmental law enforcement in Indonesia includes preventive efforts (outreach, information dissemination, formal and non-formal education) and repressive efforts (sanctions after a violation)	Situates this study’s criminal-sanction recommendations (repressive) alongside an education-based preventive complement documented in Indonesian legal scholarship
Documented non-formal practices (e.g., KLHK community outreach; village/community-level environmental outreach)	Legal/environmental outreach to communities, waste management training, environmental cadre programs, participatory workshops	Illustrates a concrete program format that has already been piloted and can be expanded to communities around the Bonded Zone in the Tangerang industrial corridor

A study of Community Learning Activity Centers (PKBM) under Law No. 20 of 2003, Article 26 paragraph (4), identifies three stages required for genuine community empowerment: the awareness stage (identifying needs genuinely felt by the community through dialogue), the knowledge-transformation stage (participatory learning, based on local potential, with a flexible schedule), and the self-reliance stage, in which the community applies and further develops what it has learned without further external encouragement. Evaluations of PKBM programs across various regions in Indonesia have found that many reach only the knowledge-transformation stage and fail to achieve genuine self-reliance ( Lukman, 2021). Non-formal environmental legal education programs for communities around the Tangerang industrial corridor should therefore be designed to pass through all three stages, including a genuine awareness/dialogue stage to identify what the community itself has already observed about waste handling in its surroundings, rather than a single one-off information session, so that the program can function as a credible complementary layer to the criminal law enforcement examined in this study. It should also be noted, as broader regulatory context, that Law No. 11 of 2020 on Job Creation has simultaneously narrowed a number of formal-procedural public participation channels in environmental decision-making, such as the community’s right to object during the Environmental Impact Assessment (Amdal) process ( Pambudhi & Ramadayanti, 2021) a shift that in fact makes community-level non-formal channels an increasingly important complement, not an increasingly irrelevant one.

## Conclusion

This comparative case study shows that Indonesian criminal courts have, in two separate and verifiable instances, succeeded in convicting corporate actors for environmental waste offenses one for domestic mismanagement of hazardous and toxic waste, the other for unlicensed cross-border waste import. Read together, these two cases illustrate the mechanism predicted by Hardin’s Tragedy of the Commons and Unequal Ecological Exchange theory, while also revealing an internal inconsistency in Indonesian sentencing practice: the larger cross-border violation which most directly embodies the international waste-trade dynamic that has drawn the greatest policy attention since 2018 received a larger fine but did not receive an equivalent remedial sanction. As Indonesia’s total ban on plastic scrap imports takes effect in 2025, the credibility of that administrative ban will depend in part on whether future criminal law enforcement can close this gap by consistently applying remedial sanctions, not merely monetary ones, to both faces of this same underlying issue, domestic and cross-border alike. Because both cases were detected solely through institutional inspection, closing this gap on the repressive side should be accompanied by a preventive step in the form of non-formal environmental legal education already mandated under Article 65 paragraph (2) of Law 32/2009 so that sanction reform and community legal education each address, respectively, the punitive and preventive faces of the same enforcement deficit.

## Ethics and consent

Ethical approval and consent were not required. This study relies exclusively on a court ruling and related enforcement records held by public institutions, together with information already published in the media; it did not involve human participants, personal data collection, or confidential information.

## Data Availability

Zenodo: Underlying data for “Disparities in Criminal Sanctions and the Role of Non-Formal Environmental Education in the Enforcement of Corporate Waste Law: A Comparative Study of Two Tangerang District Court Rulings”. https://doi.org/10.5281/zenodo.21640791 Azlina, I.S., Pratama, A.A., Hariyanto, M., Hasan, R., Ramadhan, P., Gustiara, N., Ramadhan, N.F. (2026). Data for: Disparities in Criminal Sanctions and the Role of Non-Formal Environmental Education in the Enforcement of Corporate Waste Law: A Comparative Study of Two Tangerang District Court Rulings [Data set]. Zenodo. https://doi.org/10.5281/zenodo.21640791 This project contains the following underlying data:
• Data file 1. Verbatim archive of Tangerang District Court Ruling No. 1834/Pid.Sus/2020/PN Tng (PT Indonesia Toray Synthetics), obtained from the Supreme Court Ruling Directory (primary source; court rulings are not subject to copyright under Indonesian law). • Data files 2–5. Full original-language text (with English paraphrase for accessibility) and full citation (including archived permalinks) of four mutually corroborating secondary sources concerning Tangerang District Court Ruling No. 1606/PID.B/LH/2022/PN TNG (PT Advance Recycle Technology): the Tangerang City District Attorney’s Office’s statement on the case outcome as republished by two independent media outlets, and two 2019 investigative reports by Mongabay Indonesia. • Data file 6. A source metadata index (URL, archived permalink, author, publication date, copyright status, and retrieval notes for each source and figure). • Data files 7–8. Uncropped, unedited parent image files for Figure 1 (Figure 1_comparative_summary.tiff ) and Figure 2 (Figure 2_enforcement_flow.tiff ), at full resolution and print quality (300dpi, RGB). Data are available under the terms of the Creative Commons Attribution 4.0 International license (CC-BY 4.0). Data file 1 and Data files 7–8 reproduce, respectively, a court ruling (excluded from copyright protection under Indonesian Copyright Law, Law No. 28 of 2014, Article 42 letter d) and author-generated figures; the full-text news excerpts in Data files 2–5 are reproduced under quotation/citation allowances for scholarly, non-commercial verification purposes, with full attribution to the original publishers. Zenodo: Extended data for “Disparities in Criminal Sanctions and the Role of Non-Formal Environmental Education in the Enforcement of Corporate Waste Law: A Comparative Study of Two Tangerang District Court Rulings”. https://doi.org/10.5281/zenodo.21640791 Azlina, I.S., Pratama, A.A., Hariyanto, M., Hasan, R., Ramadhan, P., Gustiara, N., Ramadhan, N.F. (2026). Data for: Disparities in Criminal Sanctions and the Role of Non-Formal Environmental Education in the Enforcement of Corporate Waste Law: A Comparative Study of Two Tangerang District Court Rulings [Data set]. Zenodo. https://doi.org/10.5281/zenodo.21640791 This project contains the following extended data:
•
case_coding_matrix.csv: a structured, researcher-generated coding of case facts against the articles of Law No. 32/2009 and against the Tragedy of the Commons/Unequal Ecological Exchange framework, underlying Table 2 and Figures 1-2 of this article. Data are available under the terms of the Creative Commons Attribution 4.0 International license (CC-BY 4.0), as this file consists entirely of the authors’ own analytical coding. No reporting guideline checklist applies to this study, as it is neither a clinical trial or protocol, a systematic review or protocol, nor an in vivo experiment.
