# Supplementary material for: Perbedaan Sanksi Pidana dan Peran Pendidikan Lingkungan Non-Formal dalam Penegakan Hukum Pengelolaan Sampah Korporasi: Studi Perbandingan Dua Putusan Pengadilan Negeri Tangerang
Source: F1000Res. 2026 Oct 7;15:1297. Originally published 2026 Aug 5. [Version 2] doi: 10.12688/f1000research.187336.2 (PMC13728441; doi:10.12688/f1000research.187336.2)
Supplement: Peer review history

# Reviewer report: Reviewer response for version 2

**DOI:** 10.5256/f1000research.209196.r529040

**Published:** 2026-11-10

**Authors:** Badal Mavry, Sneha Lohar

**Affiliations:** 1 Department of Forensic Science, IILM University (Ringgold ID: 250154), Greater Noida, Uttar Pradesh, India; 2 IILM University, Greater Noida, Uttar Pradesh, India

PEER REVIEW REPORT

 Article: Azlina IS, Pratama AA, Hariyanto M, et al. Perbedaan Sanksi Pidana dan Peran Pendidikan Lingkungan Non-Formal dalam Penegakan Hukum Pengelolaan Sampah Korporasi: Studi Perbandingan Dua Putusan Pengadilan Negeri Tangerang [version 2]. F1000Research 2026, 15:1297. https://doi.org/10.12688/f1000research.187336.2

 Article type: Case Study

 Version reviewed: Version 2 (published 07 October 2026)

 Reviewer: Badal Mavry, Department of Forensic Science, IILM University, Greater Noida, Uttar Pradesh, India

 SUMMARY OF THE ARTICLE

 The authors compare two corporate environmental criminal cases decided by the Tangerang District Court: Ruling No. 1834/Pid.Sus/2020/PN Tng against PT Indonesia Toray Synthetics (fine of Rp300 million and a court ordered clean up of hazardous and toxic (B3) waste) and Ruling No. 1606/PID.B/LH/2022/PN TNG against PT Advance Recycle Technology (fine of Rp4 billion, reconstructed from a prosecutor's press statement and media reports). The cases are read against Law No. 32 of 2009 on Environmental Protection and Management (UUPPLH), Hardin's Tragedy of the Commons and Unequal Ecological Exchange theory. The principal comparative observation is that the case with the larger fine had no remedial sanction reported in the available sources, whereas the case with the smaller fine carried a clean up order. The authors recommend standardisation of the Article 119 remedial sanction and propose a three stage non-formal environmental legal education model directed at offending corporations. Version 2 moderates several claims in response to the two earlier reports.

 The research question is relevant, the article is clearly organised, and the authors have made a commendable effort to archive their sources and to state limitations. The comments below concern whether the evidence base is described accurately and whether it supports the central comparative observation.

 VERIFICATION PERFORMED FOR THIS REPORT

 Because this report is published with the article, I list what I checked so that authors and readers can reproduce it. I read the full article (Version 2), including Tables 1 to 3, Figures 1 and 2, and both earlier reports with the author responses. I opened the Zenodo record cited in the Data availability statement (https://doi.org/10.5281/zenodo.21640791, Version v4) and the preceding version (https://doi.org/10.5281/zenodo.21619140, Version v3), and read every text and CSV file in Version v3. I read the archived copy of the VOJ.co.id article (Wayback Machine capture of 20 July 2026, linked in Data file 2), the full text of Hardin (1968) and of Brooks et al. (2018), and checked the bibliographic records of the other cited works. The Supreme Court Ruling Directory (putusan3.mahkamahagung.go.id) and the case tracking system of the Tangerang District Court returned an access denied response from my location, so for the content of the Directory page I rely on the authors' own archived capture (Zenodo v3, file 01). My expertise lies in forensic science and environmental forensics; my comments on Indonesian statutory provisions rest on the statutory text quoted in Table 1 and on the primary sources listed above.

 MAJOR COMMENTS

 1. The evidentiary status of Case 1 is described inaccurately

 The Method states that Ruling No. 1834/Pid.Sus/2020/PN Tng was "obtained in full from the Supreme Court Ruling Directory and quoted verbatim throughout this article", the Introduction states that Case 1 is "documented from the full primary court ruling", and the Conclusion describes it as "verified from the full primary court ruling". The authors' own archive of this source (Zenodo v3, file 01_putusan_toray_1834-2020-PN-Tng.txt) states that the Directory page publishes the case metadata and the operative part of the judgment (Catatan Amar) only, that no PDF or ZIP of the judgment is attached, and that the capture does not include the legal reasoning (pertimbangan hukum), the indictment (dakwaan) or witness testimony. The same file states that this scope is stated explicitly in the Method and Limitations sections of the article, which is not the case in Version 2.

 Several statements in the Case 1 section go beyond the operative part. The characterisation of the offence as a location and procedure based violation within an already licensed waste management system is an inference from the list of seized evidence, which includes permits, and not a finding of the court. The detection route (environmental compliance monitoring by the KLHK), the description of PT Indonesia Toray Synthetics as a "foreign-affiliated producer", and the "KLHK / Customs inspection" box in Figure 2 carry no cited source.

 Requested: (a) obtain the complete judgment from the court registry, or (b) revise every statement that describes Case 1 as a full or verbatim primary ruling, including Table 2 ("Primary - verbatim ruling text"), the case coding matrix, the Introduction, the Method and the Conclusion, and either source or remove the statements listed above. If the court's reasoning is unavailable for both cases, the evidentiary asymmetry on which the article is framed is smaller than presented, and this should be stated.

 2. The corroboration of Case 2 is overstated

 The Method states that Case 2 was "cross-verified through four independent news sources to ensure internal consistency of the case number, the articles charged, and the size of the sanction", and the case coding matrix states that the case number, articles charged, fine amount and payment date were confirmed identically across all four sources. Two of the four sources (Mongabay Indonesia, 21 September 2019 and 14 October 2019) predate the ruling of 15 December 2022 and the payment of 15 February 2023, and cannot confirm any of these items. The remaining two (VOJ.co.id and Koran Pelita) report the same press statement of the Tangerang City District Attorney's Office; the authors' note in Data file 3 itself describes Koran Pelita as a republication of that statement. The verdict facts for Case 2 rest on one prosecutorial press statement reported by two outlets, supplemented by two earlier reports on the investigation stage.

 Requested: describe the source base in these terms throughout (Abstract, Method, Table 2, Data availability, coding matrix). Because the press statement refers explicitly to the operative part of the judgment, I encourage the authors to request a copy of Ruling No. 1606/PID.B/LH/2022/PN TNG from the registry or the public information officer of the Tangerang District Court under Law No. 14 of 2008 on Public Information Disclosure, and to report the outcome of that request. Advising readers to consult the registrar (Implementation limitations section) does not substitute for the authors doing so, since the central observation depends on this document.

 3. A statement in the authors' own source bears directly on the central observation and is not discussed

 The central observation is that no remedial or additional sanction was reported for Case 2. In the archived VOJ.co.id article (Data file 2), after describing the 90 day re-export obligation for B3 contaminated imports under Minister of Trade Regulation No. 31 of 2016, the prosecutor states that the company did not comply with this provision and that, for this reason, a special sanction was decided ("Makanya diputuskan sanksi khusus"). The authors' own English summary in Data file 2 renders this as a special sanction having been decided. The nature of this sanction is not specified; it may refer to the fine itself or to a non monetary order. In either case the statement is part of the authors' corroborating record, and the claim that the available sources report no additional sanction cannot stand without discussion of it.

 The comparison also considers criminal sanctions only. Mongabay Indonesia (21 September 2019; Data file 5) reports that Customs froze the Bonded Zone licence of PT ART after the import was found to lack an import approval, and that nine containers originating from three Bonded Zone companies, including PT ART, were re-exported to Australia on 19 September 2019. In an import case the principal remedial measure, return of the waste, may operate through customs and administrative law before the criminal judgment, whereas a clean up order is the natural remedy for waste left on a production site. The contrast shown in Figure 1C and Figure 2 may reflect this difference in remedial pathway rather than inconsistent application of Article 119.

 Requested: discuss the special sanction statement explicitly; establish, where possible, which PT ART containers were re-exported and which administrative measures were imposed; and revise the Abstract, Table 2, Figure 1C, Figure 2, the Theoretical interpretation and the Conclusion accordingly. The cross symbol in Figure 1C signals a confirmed absence, which is inconsistent with the "not reported" wording adopted in Version 2.

 4. The fine comparison does not account for the statutory penalty ranges

 Table 1 quotes Article 104 (maximum fine Rp3 billion, no minimum), Article 105 (fine of Rp4 billion to Rp12 billion) and Article 106 (fine of Rp5 billion to Rp15 billion). Three consequences follow.

 (a) Under an alternative indictment (dakwaan alternatif) the court selects and decides on one charge. The Results statement that the court found the corporation guilty under Article 105 "or alternatively Article 106" reproduces the charging structure reported in the press statement and not a judicial finding. The operative wording reported by Koran Pelita (Data file 3), bringing waste into the environmental media of Indonesia without a permit, corresponds to Article 69(1)(c), the prohibition referenced in Article 105. A fine of Rp4 billion lies below the Article 106 minimum and equals the Article 105 minimum, which is consistent with a conviction under Article 105.

 (b) If so, the Article 106 maximum (Rp15 billion) used in Table 1 as the benchmark for the Rp4 billion fine is not applicable, and the Abstract statement that the company was proven to have violated Articles 105/106/116/118 to 119 should identify the provision actually applied.

 (c) Expressed relative to the applicable range, the Case 1 fine equals 10% of the Article 104 maximum (Rp300 million of Rp3 billion), and the Case 2 fine equals the Article 105 statutory minimum. The thirteen fold difference in absolute fines (Table 2) is largely set by the legislature, and the court in Case 2 appears to have imposed the lowest fine permitted. Table 2 describes the difference as "roughly proportionate to the scale of the violation", yet no measure of scale is reported for either case. The authors' own sources contain scale information for Case 2 that is not used: 87 containers, 24 at the PT ART Bonded Zone facility and 63 at Tanjung Priok, and about 580 tonnes of material suspected to be B3 waste that was the subject of a separate investigation concerning one of the individual suspects (Data file 4). The outcome of that investigation is not reported.

 Requested: present each fine relative to its statutory range, report the available scale indicators, state the outcome or current status of the separate investigation, and revise the interpretation of the fine contrast accordingly.

 5. Source attribution errors affect the theoretical interpretation

 The Results attribute to Mongabay Indonesia (2019b, 21 September 2019) the statements that KLHK inspected 882 containers between April and August 2019 and that 87 containers of B3 contaminated plastic waste from Hong Kong, Spain, Canada, Australia and Japan entered illegally, and present this as national background rather than as a case fact. In the authors' archive this material appears in Mongabay Indonesia (2019a, 14 October 2019; Data file 4), where the 87 containers are those attributed to PT ART itself and the countries of origin are reported by the KLHK Director General of Law Enforcement as based on the suspects' statements. Mongabay 2019b (Data file 5) concerns national Customs figures (2,041 containers, of which 455 met the requirements) and describes the 24 PT ART containers as containing plastic pellets.

 These corrections matter for the Unequal Ecological Exchange paragraph, which uses the countries of origin as case facts while the Results section describes them as background. The B3 or non B3 character of the PT ART consignment should also be stated consistently: the VOJ headline refers to B3 waste, the KLHK statement to B3 plastic, Mongabay 2019b to plastic pellets, the trade regulation cited governs imports of non B3 waste, and the operative wording reported by Koran Pelita corresponds to Article 105 (waste) rather than Article 106 (B3 waste).

 Requested: correct the citations, describe the origin information as an allegation reported at the investigation stage, and reconcile the classification of the consignment across the Abstract, Results, Table 1 and Table 2.

 6. The cited dataset version does not contain the underlying data, and the licensing of third party text requires clarification

 The Data availability statement cites https://doi.org/10.5281/zenodo.21640791 and lists Data file 1 (ruling archive), Data files 2 to 5 (secondary sources), Data file 6 (metadata index), Data files 7 and 8 (figures) and case_coding_matrix.csv. This DOI resolves to Zenodo Version v4 (published 28 July 2026), which contains only the two figure files, LICENSE and source_metadata.csv. The ruling archive, the four source files, case_coding_matrix.csv and README are present only in Version v3 (https://doi.org/10.5281/zenodo.21619140). The description field of Version v4 also retains the Version 1 wording on an inverse relationship.

 Data files 2 to 5 are described in the article as full original language text, yet each file contains an excerpt with ellipses while stating that the source is reproduced in full. The files contain copyrighted journalistic text, as the files themselves acknowledge, while the article states that the data are available under CC-BY 4.0. Authors cannot license third party copyrighted text under CC-BY.

 Requested: deposit a single complete version (or cite a version in which all listed files are present), update the Zenodo description, describe the source files accurately as excerpts, and either obtain permission from the publishers or replace the reproduced text with full citations, archived links and short quotations, stating the licence exception in the Data availability statement as the F1000Research data policy allows for restricted material.

 7. The legal framework should be brought up to date and the applicable version identified

 (a) The article does not state the dates of the offences. The Case 1 archive gives a register date of 21 September 2020, and the seized evidence includes a laboratory report dated 29 May 2019; Mongabay 2019a reports that PT ART was suspected of importing from May to June 2019. These dates should be stated, because Law No. 32 of 2009 was amended by the Job Creation legislation (Law No. 11 of 2020, subsequently replaced through Government Regulation in Lieu of Law No. 2 of 2022, enacted as Law No. 6 of 2023). Table 1 is described as the official consolidated text but cites the 2009 text. The authors should confirm whether any of the quoted provisions, and Article 63 cited in Table 3, were amended, and indicate which version applied to each case. The reference to Law No. 11 of 2020 in the final section should be updated to the instrument currently in force.

 (b) Law No. 1 of 2023 on the Criminal Code and Law No. 20 of 2025 on the Code of Criminal Procedure came into force on 2 January 2026, together with Law No. 1 of 2026 on Penal Adjustment. Published summaries indicate that the new Code regulates corporate criminal liability and lists additional penalties for corporations that include payment of compensation, repair of the consequences of the offence, performance of neglected obligations and financing of job training; legal commentary indicates that Law No. 1 of 2026 removes special minimum penalties from statutes outside the Code. The two cases predate this framework, but the recommendations (standardisation of Article 119 sanctions and the corporate education model) are forward looking, and Version 2 was published in October 2026. The authors should analyse how this framework affects their recommendations, including whether the statutory minimum fines in Articles 105 and 106 remain in force.

 8. The theoretical interpretation and the corporate education model require closer grounding

 (a) The Theoretical interpretation places in quotation marks, as Hardin's words, the statement that the long term costs of relying on conscience alone are reason enough to reject it. This wording does not appear in Hardin (1968). The corresponding sentence, in the section titled "Pathogenic Effects of Conscience", begins "The long-term disadvantage of an appeal to conscience". A direct quotation must reproduce the source exactly with a page reference; otherwise the passage should be paraphrased without quotation marks. The term coercive device does occur in Hardin, in the discussion of taxation under mutual coercion, and the pollution section of the essay, which calls for coercive laws or taxes that make treatment cheaper than discharge, may be the more apposite passage.

 (b) Hardin argues that appeals to conscience eliminate themselves over time and recommends mutual coercion. The article relies on Hardin to justify the clean up order and then proposes awareness based education as a complement. The two positions can be reconciled, since Hardin acknowledges a role for continually renewed education, but the article should address this tension explicitly.

 (c) The Toray waste was stored within the company's own premises, adjacent to licensed storage areas. To support the reading of Case 1 as pollution of a commons, the article should state into which environmental medium the court found the waste to have been dumped; otherwise the shared space premise is asserted rather than shown.

 (d) The corporate education model is presented as operationalising Article 119(d), which concerns an obligation to carry out what was unlawfully neglected. A training and audit programme is not evidently an obligation that the offender neglected, and no doctrinal or judicial authority is cited for this reading. The authors should provide such authority or anchor the proposal in another provision (see comment 7b).

 (e) The paragraph on community learning centres states that evaluations of PKBM programmes across various regions of Indonesia found that many reach only the knowledge transformation stage, citing Lukman (2021). Lukman (2021) is a qualitative study of a single centre (PKBM Tiara Dezzy, Samarinda), which reports that this centre did not conduct the awareness stage and had reached only the knowledge transformation stage. The statement should be limited to what that study found, or supported by further sources.

 (f) The appeal to deterrence theory in the Theoretical interpretation carries no citation.

 MINOR COMMENTS

 1. The title is in Indonesian, while the text and the Zenodo dataset title ("Disparities in Criminal Sanctions and the Role of Non-Formal Environmental Education in the Enforcement of Corporate Waste Law: A Comparative Study of Two Tangerang District Court Rulings") are in English. The F1000Research article guidelines require articles to be written in English; an English title consistent with the dataset title is needed.

 2. Introduction: Brooks et al. (2018) report that China alone accounted for 45.1% of cumulative global plastic waste imports since 1992, and China and Hong Kong together for 72.4%. The article attributes 72.4% to China alone and describes it as a share of plastic traded globally rather than of cumulative imports.

 3. The Ismawati et al. (2024) chapter appears in Gündoğdu S (ed.), Plastic Waste Trade: A New Colonialist Means of Pollution Transfer, Springer, 2024 (https://doi.org/10.1007/978-3-031-51358-9_9), not in a volume edited by Bank MS titled Plastic in the Environment: Patterns and Processes. I could not access the full chapter; please give the page for the 141% figure.

 4. The Kanun article on environmental law enforcement under Law No. 32 of 2009 that I could locate is by Sodikin, Kanun: Jurnal Ilmu Hukum, Vol. 12, No. 3 (2010), and not by Solikin (2019). Please verify the author, year, volume and pages.

 5. The archived VOJ.co.id page is dated 16 February 2023; the reference list gives 15 February 2023, which is the date of payment.

 6. The Amendments box states that Figures 1 and 2 are unchanged, whereas the response to the first report states that the Figure 1 and 2 captions and source notes were revised. Please reconcile. The source lines of both figures read "constructed by the author" for an article with seven authors.

 7. Figure 1A places Case 1 only at its verdict; adding the offence or detection date would make the two timelines comparable. In Figure 2 the freezing of the Case 2 Bonded Zone licence appears in the diagram but not in the text.

 8. The Theoretical interpretation refers to a Bonded Zone facility established just weeks after China's National Sword policy, whereas the Results state that the company was established in January 2018 and obtained Bonded Zone status in March 2019. Please correct.

 9. Conclusion: the plastic scrap import ban is said to take effect in 2025. The ban has applied since 1 January 2025; the tense should be revised.

 10. The recommendation to reverse the burden of proof at the border should be discussed against the existing requirement for an import approval and a KLHK recommendation under Minister of Trade Regulation No. 31 of 2016, and against the 2025 ban, under which, according to official statements, no import recommendations are issued.

 11. The funding statement repeats the sentence on the role of the funders, and the Acknowledgment again acknowledges LPDP support, which the article guidelines ask to be stated only under Grant information.

 RESPONSES TO THE F1000RESEARCH CASE STUDY QUESTIONS

 Is the background of the case's history and progression described in sufficient detail?

 Partly

 The context after 2018 is well described. The offence dates of both cases, the detection route for Case 1, the scale indicators and parallel administrative measures for Case 2, and the outcome of the separate investigation are missing (comments 1, 3, 4 and 7a).

 Is the work clearly and accurately presented and does it cite the current literature?

 No

 The article is clearly written, but the evidentiary status of Case 1, the corroboration of Case 2, several source attributions and one direct quotation are presented inaccurately (comments 1, 2, 5 and 8a; minor comments 2 to 4), and the legal framework in force since 2023 and 2026 is not considered (comment 7).

 If applicable, is the statistical analysis and its interpretation appropriate?

 Not applicable

 Are all the source data underlying the results available to ensure full reproducibility?

 No

 The cited dataset version does not contain the source files, the full text of neither judgment is available, and the licence statement conflicts with the third party content of the files (comments 1, 2 and 6).

 Are the conclusions drawn adequately supported by the results?

 No

 The central observation, that a remedial sanction was present in Case 1 and absent in Case 2, rests on an absence in a single press statement that itself reports an unspecified special sanction, does not consider administrative remediation in the import case, and interprets a fine difference largely set by the statutory ranges (comments 3 and 4).

 Is the case presented with sufficient detail to be useful for teaching or other practitioners?

 Partly

 The comparison of a domestic hazardous waste case with a transboundary import case is instructive, and the archived sources are a useful resource. Its value to practitioners depends on the corrections requested above.

 APPROVAL STATUS

 Recommended status

 Not Approved

 The concerns relate to the accuracy with which the evidence is described and to whether that evidence supports the central comparative observation; the research question itself is worthwhile. The concerns can be resolved. Obtaining the two judgments, or describing the actual source base accurately and addressing the special sanction statement, the administrative remediation in Case 2 and the statutory fine ranges, would allow the comparison to be assessed on a sound footing. I would be glad to review a revised version.

 Competing interests: No competing interests were disclosed.

 Reviewer expertise: Forensic science; environmental forensics; evaluation of documentary and physical evidence.

Is the case presented with sufficient detail to be useful for teaching or other practitioners?

Partly

Is the work clearly and accurately presented and does it cite the current literature?

No

If applicable, is the statistical analysis and its interpretation appropriate?

Not applicable

Are all the source data underlying the results available to ensure full reproducibility?

No

Are the conclusions drawn adequately supported by the results?

No

Is the background of the case’s history and progression described in sufficient detail?

Partly

Reviewer Expertise:

Forensic science; environmental forensics; evaluation of documentary and physical evidence.

We confirm that we have read this submission and believe that we have an appropriate level of expertise to state that we do not consider it to be of an acceptable scientific standard, for reasons outlined above.

# Reviewer report: Reviewer response for version 2

**DOI:** 10.5256/f1000research.209196.r527897

**Published:** 2026-11-02

**Authors:** Arief Fahmi Lubis

**Affiliations:** 1 Sekolah Tinggi Hukum Militer, Jakarta, Indonesia

After reviewing the revisions, I am pleased to confirm that I approve the revised version of the manuscript. I believe the authors have adequately addressed my previous comments and concerns. Therefore, I am happy to change my previous status from "Approved with Reservations" to "Approved".

Is the case presented with sufficient detail to be useful for teaching or other practitioners?

Partly

Is the work clearly and accurately presented and does it cite the current literature?

Partly

If applicable, is the statistical analysis and its interpretation appropriate?

Not applicable

Are all the source data underlying the results available to ensure full reproducibility?

Yes

Are the conclusions drawn adequately supported by the results?

Partly

Is the background of the case’s history and progression described in sufficient detail?

Yes

Reviewer Expertise:

Environmental Law, Criminal Law, Corporate Criminal Liability, and Environmental Law Enforcement.

I confirm that I have read this submission and believe that I have an appropriate level of expertise to confirm that it is of an acceptable scientific standard.

# Reviewer report: Reviewer response for version 2

**DOI:** 10.5256/f1000research.209196.r514125

**Published:** 2026-10-15

**Authors:** Muhammad Natsir

**Affiliations:** 1 Universitas Samudra, Langsa, Aceh, Indonesia

I have now assessed the recent revisions and the authors' response to my previous report (Approved with Reservations).

 I can confirm that the authors have addressed all the points raised in my review:

 1. The Abstract has been appropriately revised, replacing the claim of an "inverse relationship" with "observed contrast", which accurately reflects the two-case scope of this study.

 2. The manuscript now consistently distinguishes between the two cases throughout the Introduction, Method, Results, Discussion and Conclusion - Case 1 (PT Indonesia Toray Synthetics No. 1834/Pid.Sus/2020/PN Tng) as a verbatim primary source, and Case 2 (PT Advance Recycle Technology No. 1606/PID.B/LH/2022/PN TNG) as a reconstructed secondary source. This was my main concern and it is now fully resolved.

 3. The theoretical discussion on Unequal Ecological Exchange and the role of non-formal environmental education is now correctly framed as an interpretive and policy hypothesis, rather than as a direct empirical finding. The addition of the concrete three-stage non-formal education model is a valuable improvement and provides the novel policy contribution requested.

 4. Table 1 and Table 2 wording revisions are consistent and appropriate. The existing Zenodo DOI remains valid.

 Given these comprehensive revisions, I am happy to amend my previous status.

 My recommendation for this revised version is: APPROVED

 No further revisions are required from my side.

Is the case presented with sufficient detail to be useful for teaching or other practitioners?

Yes

Is the work clearly and accurately presented and does it cite the current literature?

Yes

If applicable, is the statistical analysis and its interpretation appropriate?

I cannot comment. A qualified statistician is required.

Are all the source data underlying the results available to ensure full reproducibility?

Yes

Are the conclusions drawn adequately supported by the results?

Yes

Is the background of the case’s history and progression described in sufficient detail?

Yes

Reviewer Expertise:

Environmental Law, Criminal Law, Environmental Criminal Law

I confirm that I have read this submission and believe that I have an appropriate level of expertise to confirm that it is of an acceptable scientific standard.

# Reviewer report: Reviewer response for version 1

**DOI:** 10.5256/f1000research.206763.r509403

**Published:** 2026-08-19

**Authors:** Muhammad Natsir, Zaki Ulya, S.H., M.H.

**Affiliations:** 1 Universitas Samudra, Langsa, Aceh, Indonesia

1. Comment: The background is presented appropriately and clearly demonstrates the research gap regarding the disparity of sanctions.

 2. Comment: The manuscript is presented well and refers to Law No. 32 of 2009 on Environmental Protection and Management. The literature cited is adequate and relevant to the topic.

 3. Comment: I am unable to select from the available options as I did not find any statistical analysis in this article. Legal research articles rarely use statistics.

 4. Comment: The main source data, namely the court decisions, can be accessed by anyone through the Supreme Court’s Decision Directory.

 5. Comment: The conclusions are appropriate and are supported by the comparative analysis of the court decisions.

 6. Comment: This case is very useful for enhancing knowledge of environmental criminal law and for legal enforcement practice. This article makes a very significant contribution to environmental criminal law and is worthy of indexing after minor revisions.

 Additional Minor Suggestions: Elaborate on the limitations of the study. The results of 2 cases cannot be generalized. Add a concrete model of non-formal education for offending corporations as a specific form of novelty. Overall Recommendation: Accept with minor revisions

Is the case presented with sufficient detail to be useful for teaching or other practitioners?

Yes

Is the work clearly and accurately presented and does it cite the current literature?

Yes

If applicable, is the statistical analysis and its interpretation appropriate?

I cannot comment. A qualified statistician is required.

Are all the source data underlying the results available to ensure full reproducibility?

Yes

Are the conclusions drawn adequately supported by the results?

Yes

Is the background of the case’s history and progression described in sufficient detail?

Yes

Reviewer Expertise:

Environmental Law, Criminal Law, Environmental Criminal Law

We confirm that we have read this submission and believe that we have an appropriate level of expertise to confirm that it is of an acceptable scientific standard, however we have significant reservations, as outlined above.

# Response

**Published:** 2026-08-23

**Authors:** Ira Sinta

**Affiliations:** Gadjah Mada University Faculty of Law, Yogyakarta, Special Region of Yogyakarta, Indonesia

We thank the reviewers for their positive assessment and constructive suggestions. We have revised the manuscript (Version 2) to address both points raised.

 1. Elaboration of limitations regarding generalizability: We have expanded the "Implementation limitations and recommendations" section to state explicitly that, as a comparison of only two cases, this study's findings depict a pattern worth further investigation rather than a statistically representative or generalizable sample of Indonesian environmental sentencing practice. We have also moderated related claims throughout the Abstract, Results, Discussion, and Conclusion (e.g., changing "inverse relationship" to "observed contrast" and adding qualifiers such as "across the two cases examined") to avoid overstating what a two-case comparison can support, consistent with feedback also raised independently by another reviewer.

 2. Concrete model of non-formal education for offending corporations: We have added a new proposal in the "Non-formal environmental legal education as a preventive complement" section: a three-stage "Corporate Environmental Compliance Education Program" directed specifically at offending corporations (rather than surrounding communities), consisting of (1) an awareness stage (mandatory joint disclosure session with KLHK/community representatives), (2) a knowledge-transformation stage (certified compliance training on the specific statutory provisions violated), and (3) an institutionalization stage (establishing an internal compliance unit, verified by a follow-up KLHK audit). This model operationalizes Article 119(d)'s "obligation to carry out what was neglected" and is explicitly framed as a policy recommendation rather than a claim tested against the two cases examined.

 We believe these revisions strengthen the manuscript's methodological caution and its contribution to practice. We are grateful for the reviewers' recommendation and remain happy to make further changes if needed.

# Reviewer report: Reviewer response for version 1

**DOI:** 10.5256/f1000research.206763.r509411

**Published:** 2026-08-18

**Authors:** Arief Fahmi Lubis

**Affiliations:** 1 Sekolah Tinggi Hukum Militer, Jakarta, Indonesia

This case study examines differences in criminal sanctions and the potential role of non-formal environmental legal education in the enforcement of corporate waste law in Indonesia. The authors compare two environmental criminal cases decided by the Tangerang District Court: PT Indonesia Toray Synthetics, involving the disposal of hazardous and toxic waste (B3) without a permit, and PT Advance Recycle Technology, involving unlicensed cross-border waste imports. The analysis is conducted using a qualitative documentary case-study approach and is framed by Law No. 32 of 2009, Hardin’s Tragedy of the Commons, and the theory of Unequal Ecological Exchange. The article further proposes that non-formal environmental legal education could complement repressive criminal enforcement, particularly because both cases were detected through institutional inspection rather than community reporting.

 The manuscript addresses an important and relevant issue in Indonesian environmental law, particularly the relationship between corporate criminal liability, remedial sanctions, illegal waste management/importation, and preventive environmental education. The comparison of domestic hazardous-waste mismanagement and cross-border waste importation is potentially valuable.

 Overall, the manuscript is clearly structured and provides useful documentary and legal analysis. However, several aspects require clarification or qualification before the conclusions can be considered fully supported. The most important issue concerns the evidentiary basis of the second case. Unlike the first case, for which the authors obtained and quoted the full primary court ruling, the second case is reconstructed from a prosecutor's statement and four secondary media sources because the full court ruling could not be obtained. The authors appropriately acknowledge this limitation, but some statements in the manuscript nevertheless appear broader than the available evidence permits.

 Assessment of the Review Questions

 1. Is the background of the case’s history and progression described in sufficient detail?

**Yes.**

 The manuscript provides a sufficiently detailed account of the broader background and the progression of both cases. The Introduction establishes the post-2018 international waste-trade context and explains the relevance of China's National Sword policy. It then introduces the two cases and explains their comparative significance.

 The case sections provide useful information concerning the factual circumstances, legal provisions, institutional investigations, charges, court outcomes, fines, and remedial sanctions. The first case is particularly well documented because it is based on the full court ruling.

 The second case is also described in reasonable detail, although its evidentiary basis is necessarily weaker because the full judicial reasoning is unavailable. This limitation is appropriately acknowledged by the authors.

 2. Is the work clearly and accurately presented and does it cite the current literature?

**Partly.**

 The manuscript is generally clear, well organized, and appropriately referenced. It cites relevant foundational literature, including Hardin, Martínez-Alier, Hornborg, and more recent work concerning Indonesia's waste trade. It also cites the relevant Indonesian legislation and documentary sources.

 However, several claims would benefit from more precise qualification and additional supporting literature.

 First, the manuscript states that Indonesian legal scholarship has “largely focused” on administrative/civil liability or the Tragedy of the Commons, and that empirical case studies based on verifiable primary rulings remain scarce. These are potentially important research-gap claims, but they require stronger evidentiary support from the literature. The authors should explain more systematically how the existing literature was identified and why it supports the characterization of the research gap.

 Second, the manuscript repeatedly emphasizes “verifiable primary rulings,” while the second case is explicitly based on secondary sources. This creates a tension between the stated research objective and the actual evidence base. The authors should make this distinction explicit throughout the manuscript and avoid presenting both cases as having the same evidentiary status.

 Third, the theoretical interpretation of the Advance Recycle Technology case through Unequal Ecological Exchange relies partly on facts such as foreign management and the origins of waste from several countries. These observations are potentially relevant, but the authors should distinguish clearly between facts established by documentary evidence and theoretical interpretation.

 I therefore recommend that the authors strengthen the literature review supporting the claimed research gap and more consistently distinguish primary evidence from secondary evidence.

 3. If applicable, is the statistical analysis and its interpretation appropriate?

**Not applicable.**

 The study is explicitly designed as a qualitative legal case study using documentary analysis. No statistical analysis is required for the research design presented.

 4. Are all the source data underlying the results available to ensure full reproducibility?

**Yes.**

 The authors have made substantial efforts to provide the underlying materials through Zenodo. These include the full text of the first court ruling, the secondary sources used to reconstruct the second case, source metadata, figures, and the researcher-generated case-coding matrix.

 However, the availability of these materials does not eliminate the evidentiary limitation concerning the second case. The full judicial reasoning remains unavailable. The authors should therefore continue to describe the second case as a secondary-source reconstruction rather than implying that its evidentiary status is equivalent to the first case.

 5. Are the conclusions drawn adequately supported by the results?

**Partly.**

 The central findings concerning the different fines and the presence/absence of a recorded remedial sanction are supported by the documentary material presented. The comparison shows that the Toray case resulted in a Rp300 million fine together with a clean-up obligation, while the Advance Recycle Technology case resulted in a Rp4 billion fine with no additional remedial sanction reported in the available sources.

 However, the manuscript should be more cautious in characterizing these two cases as demonstrating an “inverse relationship” between fine size and remedial sanctions. With only two cases, this observation is best described as a finding or pattern observed in the two cases rather than an established relationship in Indonesian environmental sentencing generally.

 The authors themselves recognize this limitation, stating that the two cases do not constitute a statistically representative sample and that a larger multi-case study would be needed.

 I recommend that the wording of the Results, Discussion, Abstract, and Conclusion be revised consistently to reflect this limitation. For example, the authors could refer to an “observed contrast” or “case-specific pattern” rather than an “inverse relationship” where the latter could imply a broader empirical relationship.

 The conclusion that Article 119 is applied inconsistently should likewise be qualified as an observation from the two cases examined, rather than a general conclusion concerning Indonesian environmental adjudication.

 Points That Must Be Addressed

 Major Comment 1: Evidentiary status of the second case

 This is the most important issue.

 The first case is supported by a full primary court ruling, whereas the second case is reconstructed from a prosecutor's statement and four media sources. The authors explicitly acknowledge that they could not obtain the complete primary ruling and that the judicial reasoning is therefore unavailable.

 This limitation is scientifically important because the article's principal comparison concerns the criminal liability and sanctions imposed by the courts. Without the full reasoning of the second judgment, it is difficult to independently verify why the court imposed the particular sanction and, especially, why no additional remedial sanction was imposed.

 The authors should therefore:

1. Clearly label all findings concerning Case 2 as based on secondary-source reconstruction.
2. Avoid suggesting that the two cases have equivalent evidentiary status.
3. Where possible, provide a direct link, archived copy, or additional official source for the judgment or official court documentation.
4. Revise any conclusion that depends specifically on the absence of an Article 119 sanction to state that **no additional remedial sanction was reported in the available sources**, rather than stating categorically that the court imposed no such sanction.
5. Consider whether the comparison should be presented primarily as an exploratory two-case comparison rather than definitive evidence of inconsistency in Indonesian sentencing practice.

Major Comment 2: Strengthen the basis for the research gap

 The manuscript states that empirical case studies based on verifiable primary rulings are scarce and that non-formal environmental education has received limited attention. These are important claims, but the manuscript should provide a more transparent basis for establishing these gaps.

 The authors should explain more clearly how the existing literature was identified and why the cited literature demonstrates the claimed gap. A brief systematic description of the literature search or selection process would strengthen the justification for the study.

 Major Comment 3: Moderate the claim of an “inverse relationship”

 The manuscript states that:

 “The most striking finding from this comparison is the inverse relationship between the size of the fine and the presence of a remedial sanction.”

 With only two cases, this wording may overstate the evidentiary strength of the finding.

 The authors should revise this language throughout the manuscript. The evidence supports an **observed contrast between two cases**, but it does not establish a general relationship between fine size and remedial sanctions in Indonesian environmental criminal adjudication.

 A more cautious formulation would be:

 “The comparison reveals a notable contrast between the two cases: the case with the larger fine did not have a remedial sanction reported in the available sources, whereas the case with the smaller fine included a specific clean-up obligation.”

 This would accurately reflect the evidence without weakening the importance of the finding.

 Major Comment 4: Clarify the evidentiary basis of the environmental education argument

 The section on non-formal environmental legal education is interesting and potentially valuable. The authors correctly identify Article 65(2) and Article 63 as relevant legal foundations and connect them with community outreach and non-formal education.

 However, the empirical connection between the two criminal cases and the proposed educational intervention is relatively indirect.

 The fact that both cases were detected through institutional inspection does not by itself establish that non-formal environmental education would have enabled earlier detection or prevention. This is a plausible policy hypothesis, but it is not directly demonstrated by the two cases.

 The authors should therefore distinguish between:

- what the two cases empirically demonstrate; and
- what the authors propose as a preventive policy recommendation.

The language should be modified from a causal implication toward a clearly identified **preventive or policy hypothesis**.

 Minor Comments

1. The distinction between primary and secondary sources should be maintained consistently in the tables, figures, Results, Discussion, and Conclusion.
2. The phrase “two separate and verifiable instances” in the Conclusion should be reconsidered because the second case is not verified from the full primary judicial text.
3. The manuscript could more clearly distinguish the court's findings from the authors' interpretation, particularly in the theoretical discussion involving the Tragedy of the Commons and Unequal Ecological Exchange.
4. The authors should ensure that claims concerning the absence of an additional sanction are consistently expressed as “not reported in the available sources” where the complete judgment is unavailable.
5. The theoretical interpretation is interesting, but some statements concerning foreign ownership/management and the transfer of ecological burdens should be presented as theoretical interpretation rather than as empirically demonstrated causation.

Overall Assessment

 This is a relevant and potentially valuable case study that contributes to discussion of corporate environmental criminal liability, remedial sanctions, illegal waste imports, and preventive environmental education in Indonesia. The use of two Tangerang District Court cases provides a useful comparative framework, and the manuscript has made commendable efforts to make its underlying documentary sources available.

 The principal issue is not the relevance of the research question, but the **unequal evidentiary quality of the two cases** and the resulting need to moderate several conclusions. The first case is supported by a primary judicial source, while the second is reconstructed from secondary sources. This limitation is openly acknowledged by the authors, which is a strength, but its implications need to be reflected more consistently in the claims made throughout the article.

 The article could become scientifically sound with revision, particularly if the authors clearly distinguish primary from secondary evidence, moderate the generality of the conclusions drawn from only two cases, strengthen the justification of the research gap, and clarify that the proposed role of non-formal environmental education is a policy recommendation rather than a causal finding established by the cases.

 Subject to these revisions, I consider the study potentially suitable for indexing.

Is the case presented with sufficient detail to be useful for teaching or other practitioners?

Partly

Is the work clearly and accurately presented and does it cite the current literature?

Partly

If applicable, is the statistical analysis and its interpretation appropriate?

Not applicable

Are all the source data underlying the results available to ensure full reproducibility?

Yes

Are the conclusions drawn adequately supported by the results?

Partly

Is the background of the case’s history and progression described in sufficient detail?

Yes

Reviewer Expertise:

Environmental Law, Criminal Law, Corporate Criminal Liability, and Environmental Law Enforcement.

I confirm that I have read this submission and believe that I have an appropriate level of expertise to confirm that it is of an acceptable scientific standard, however I have significant reservations, as outlined above.

# Response

**Published:** 2026-08-23

**Authors:** Ira Sinta

**Affiliations:** Gadjah Mada University Faculty of Law, Yogyakarta, Special Region of Yogyakarta, Indonesia

We are grateful to the reviewer for this detailed and constructive assessment, which has substantially improved the manuscript. We address each point below; all changes have been made in Version 2.

 Major Comment 1 (Evidentiary status of the second case): We have revised the manuscript throughout to consistently distinguish Case 2 as a secondary-source reconstruction. Specifically: (a) the Introduction, Research objective, and Method sections now state explicitly that the two cases are of "deliberately unequal evidentiary status" and that this study is "best characterized as an exploratory two-case comparison rather than a matched pair of equivalently verified rulings"; (b) the opening of the Case 2 results section now states directly that findings there "should be read as the authors' reconstruction of the case outcome rather than an independently verified account of the court's reasoning"; (c) we have added a sentence noting that all four secondary sources were preserved as archived Wayback Machine permalinks at the time of retrieval (recorded in the underlying data on Zenodo) to support long-term verifiability, since the full court ruling itself remains unavailable through the public directory; (d) we have revised every statement about the absence of an additional sanction in Case 2 to read "not reported in the available sources" rather than implying a confirmed negative finding, including in Table 2, the Case 2 narrative, the Theoretical interpretation, and the Conclusion; and (e) the Implementation limitations section now explicitly recommends that readers seeking the court's own reasoning consult the Tangerang District Court registrar directly.

 Major Comment 2 (Basis for the research gap): We have added a sentence in the Problem statement explaining that the characterization of the literature gap is based on the authors' review of Indonesian environmental law scholarship in national journals (including Kanun Journal of Legal Studies and the Indonesian Journal of Environmental Law) and postgraduate legal research over the past decade, while explicitly acknowledging that this was not a formal systematic literature search and that the characterization should be read as indicative rather than exhaustive.

 Major Comment 3 (Moderating the "inverse relationship" claim): We have adopted wording very close to the reviewer's suggested formulation throughout the manuscript (Abstract, Theoretical interpretation, Conclusion), replacing "inverse relationship" with "observed contrast" and explicitly noting that this is "an observation drawn from these two cases rather than an established relationship in Indonesian environmental sentencing generally," with a note that a larger, systematically sampled multi-case study would be required to establish whether the pattern holds more generally.

 Major Comment 4 (Evidentiary basis of the environmental education argument): We have added an explicit statement in the "Non-formal environmental legal education" section clarifying that the link between institutional-inspection-only detection and the proposed educational intervention "is a policy hypothesis rather than a causal finding empirically demonstrated by the two cases," and that the cases show only that detection occurred through institutional inspection, not that non-formal education would have enabled earlier detection. The same qualification has been added to the Conclusion and to the description of the new corporate education model proposed in response to another reviewer's suggestion.

 Minor Comments:

 - We have reviewed the tables, figures, Results, Discussion, and Conclusion and revised the language to consistently maintain the primary/secondary source distinction (e.g., Table 2's "Source status" row, Figure 1/2 captions and source notes, and in-text attributions).

 - We have revised the phrase "two separate and verifiable instances" in the Conclusion to "two documented instances of unequal evidentiary weight one verified from the full primary court ruling, the other reconstructed from corroborating secondary sources."

 - We have added a clarifying sentence in the Unequal Ecological Exchange discussion noting that this theory "is applied here as an interpretive lens for reading the case-level facts, not as an independently verified causal mechanism," and revised the Case 2 theoretical interpretation to describe foreign management and waste origins as "documented facts" that are "consistent with, though they do not on their own empirically establish," the UEE framework.

 - All statements concerning the absence of an additional sanction have been revised to read "not reported in the available sources" or equivalent, consistently.

 We believe these revisions directly address the central concern identified by the reviewer the unequal evidentiary quality of the two cases while preserving the comparative structure and contribution of the study. We thank the reviewer again for a thorough and genuinely constructive review, and remain happy to make further changes if needed.
